# Auto-Antibody Production During Experimental Atherosclerosis in *ApoE^-/-^* Mice

**DOI:** 10.3389/fimmu.2021.695220

**Published:** 2021-07-09

**Authors:** Mark A. Hutchinson, Han-Sol Park, Kimberly J. Zanotti, Juan Alvarez-Gonzalez, Jing Zhang, Li Zhang, Richard Telljohann, Mingyi Wang, Edward G. Lakatta, Patricia J. Gearhart, Robert W. Maul

**Affiliations:** ^1^ Laboratory of Molecular Biology and Immunology, National Institute on Aging, NIH, Baltimore, MD, United States; ^2^ Laboratory of Cardiovascular Science, National Institute on Aging, NIH, Baltimore, MD, United States

**Keywords:** AID, atherosclerosis, B cells, antibodies, antigens

## Abstract

Current models stipulate that B cells and antibodies function during atherosclerosis in two distinct ways based on antibody isotype, where IgM is protective and IgG is inflammatory. To examine this model, we generated *ApoE^-/-^ Aid^-/-^* mice, which are unable to produce IgG antibodies due to the absence of activation-induced deaminase (AID) but maintain high plasma cholesterol due to the absence of apolipoprotein E (APOE). We saw a dramatic decrease in plaque formation in *ApoE^-/-^ Aid^-/-^* mice compared to *ApoE^-/-^* mice. Rigorous analysis of serum antibodies revealed both *ApoE^-/-^* and *ApoE^-/-^ Aid^-/-^* mice had substantially elevated titers of IgM antibodies compared to C57BL/6J controls, suggesting a more complex dynamic than previously described. Analysis of antigen specificity demonstrated that *ApoE^-/-^ Aid^-/-^* mice had elevated titers of antibodies specific to malondialdehyde-oxidized low density lipoprotein (MDA-oxLDL), which has been shown to block macrophage recruitment into plaques. Conversely, *ApoE^-/-^* mice showed low levels of MDA-oxLDL specificity, but had antibodies specific to numerous self-proteins. We provide evidence for a hierarchical order of antibody specificity, where elevated levels of MDA-oxLDL specific IgM antibodies inhibit plaque formation. If the level of MDA-oxLDL specific IgM is insufficient, self-reactive IgM and IgG antibodies are generated against debris within the arterial plaque, resulting in increased inflammation and further plaque expansion.

## Introduction

During atherosclerosis, low density lipoproteins are oxidized (oxLDL) and activate endothelial cells to release cytokines, which then recruit circulating monocytes to invade the arterial wall and uptake oxLDL (1). These inflammatory signals also promote the recruitment of other innate and adaptive immune cells to the plaque (2). This includes B cells, which have been identified in the adventitia and surrounding adipose tissue (3). Depletion of total B cells has been shown to alter atherosclerosis in mouse models (4, 5), leading to proposals that different B cell subsets can either prevent or promote disease. The introduction of B-1 cells into B cell-deficient mice decreased plaque and inflammation (6, 7), presumably by secreting IgM antibodies that bind to oxLDL (8). IgM antibodies could prevent disease by sequestering oxLDL away from macrophages and inhibiting plaque initiation (9–11). In contrast, depletion of the more abundant B-2 cells decreased atherosclerosis (12, 13), suggesting that B-2 cells and their antibodies are inflammatory. Upon activation, B-2 cells form germinal centers and fine-tune their antigen binding through somatic hypermutation and class switch recombination to produce high affinity IgG antibodies. Disruption of germinal center generation substantially decreased atherosclerosis (14, 15). Although B-2 cells express both IgM and IgG, the IgG isotype may bind to atherosclerotic antigens and attach to Fc*γ* receptors on macrophages and vascular smooth muscle cells to increase foam cell development and initiate hyperplasia, respectively (16–20).

While cellular manipulation indicated a demarcation between B-1 and B-2 cells in inhibiting or promoting atherosclerosis, the mechanisms could simply be ascribed to the isotypes and specificities of antibodies. For example, mice deficient in the ability to produce high levels of serum IgM (*sIgM^-/-^* or *Xbp1^-/-^*) had increased atherosclerosis (21–23). However, this may be more complicated, as different plasma cell-deficient models (*Prdm1^-/-^*) reduced circulating antibodies but decreased plaque (15, 20). Direct examination of antibody function using antibody transfer or immunization also produced inconsistent findings. Treatment of mice with anti-phosphorylcholine-specific IgM diminished plaque development (24), and treatment with IgG from *ApoE^-/-^* mice boosted atherosclerosis (15, 20), supporting the theory that IgM is helpful and IgG is harmful. However, immunization with either plaque material or malondialdehyde-oxidized LDL (MDA-oxLDL) significantly prevented atherosclerosis by boosting IgG but not IgM titers (25, 26). These numerous conflicting reports raise essential questions about the role of antibody isotypes and antigen specificity during atherosclerosis.

To address this controversy, we generated double-knockout mice which are deficient in both apolipoprotein E (ApoE) and activation-induced deaminase (AID) to rigorously analyze the function of antibody isotypes. The APOE protein is a component of very-low density lipoprotein (VLDL) and is important for proper binding of VLDL to the LDL receptor in the liver. ApoE deficiency results in elevated levels of serum cholesterol, which is exacerbated by a high-fat diet. AID is a cytosine DNA deaminase expressed in activated B cells to initiate somatic hypermutation and class switch recombination of antibodies (27). Thus, AID deficiency results in a complete block in the production of IgG, IgE, and IgA classes of antibodies, which allows for a direct examination of antibody isotype on atherosclerosis. Additionally, we performed a comprehensive analysis of B cell and antibody identification to address the effects of atherosclerosis on B cell function. Compared to *ApoE^-/-^* mice, we found that *ApoE^-/-^ Aid^-/-^* mice displayed an 80% reduction in lipid content on the aorta while retaining high levels of serum cholesterol. Meanwhile, examination of antibody concentrations revealed that *ApoE^-/-^* mice have a 2.8-fold and 6-fold increase in IgM and IgG1 titers respectively, while *ApoE^-/-^ Aid^-/-^* mice had a 4-fold increase in IgM compared to C57BL/6J mice. Surprisingly, the antibodies from *ApoE^-/-^ Aid^-/-^* mice showed specificity to malondialdehyde (MDA)-oxLDL, while *ApoE^-/-^* mice displayed low levels of MDA-oxLDL binding. Instead, using protein array screening, we identified that the high IgM and IgG1 titers in *ApoE^-/-^* mice bound numerous self-proteins. Thus, our results convincingly demonstrate that MDA-oxLDL-specific IgM had a striking effect on inhibiting plaque development. If MDA-oxLDL-specific IgM titers are insufficient, plaque accumulation promotes the generation of autoantibodies driving inflammation and defines a distinct role for antigen specificity during the development and progression of atherosclerosis. Our data support the hypothesis that immunization strategies promoting oxLDL-specific antibody responses will prevent atherosclerosis development.

## Materials and Methods

### Mice

C57BL/6J and *ApoE^-/-^* (B6.129P2-*Apoe^tm1Unc^*/J) mice were purchased from The Jackson Laboratory. *ApoE^-/-^ Aid^-/-^* double-knockout mice were generated in our mouse colony. Male mice were raised on chow diet until they reached full adult maturity at 12 weeks of age and switched to high-fat diet (#D12079B, Research Diets Inc., 21% fat and 0.15% cholesterol) until 28 weeks of age to exacerbate plaque formation. Only male mice were used to eliminate any effects of sex on disease severity (28). Cholesterol levels were measured from animals that fasted overnight using an HDL and LDL/VLDL Quantitation Kit (Sigma) per manufacturer’s recommendations. All animal protocols were reviewed and approved by the Animal Care and Use Committee of the National Institute on Aging.

### Histology

For Oil Red O staining, freshly dissected intact aortas were fixed with 4% paraformaldehyde. Samples were mixed with propylene glycol to remove water and incubated with 0.5% Oil Red O in propylene glycol solution (Sigma Aldrich). Excess stain was washed with 85% propylene glycol, and stained aortas were imaged with a Leica M165 FC microscope. The staining area was calculated using HALO software (Indica Labs). Aortas were fixed with 4% paraformaldehyde and set in paraffin blocks. Samples were then sectioned and stained with hematoxylin and eosin, or Verhoeff-Van Gieson by AML Laboratories. Images were generated using a BZ-X800 microscope (Keyence).

### Flow Cytometry

Spleen cells were obtained by mechanical separation followed by lysis of red blood cells and centrifugation. Lymphocytes were isolated from the peritoneal cavity by exposing the membrane and injecting 5 mL of RPMI 1640 media (Thermo Fisher) into the cavity. The fluid was then collected and centrifuged to obtain lymphocytes. The aorta and its perivascular fat were collected from mice through manual isolation. The tissue was mechanically separated and enzymatically digested [0.8 mg/mL Dispase (Gibco), 0.2 mg/mL Collagenase IA (Sigma), 0.2 mg/mL Collagenase XI (Sigma), and 10 U/mL DNase I (Ambion)] for 20 mins at 37°C to isolate lymphocytes. For analysis, cells were treated with TruStain FcX™ block (Biolegend) and stained using the following antibody panels from Biolegend except where noted. Total B cells: CD19^+^ (clone 6D5); germinal center B cells: B220^+^ (clone RA3-6B2), CD38^-^ (clone 90) and GL7^+^ (clone GL-7); follicular B cells: CD19^+^, GL7^-^, CD3e^-^ (clone 145-2C11), GR-1^-^ (clone RB6-8C5), CD93^-^ (clone AA4.1), CD138^-^ (clone 281-2), CD43^-^ (clone S7 from BD Pharmingen), F4/80^-^ (clone BM8), CD21^lo^ (clone 7E9) and CD23^+^ (clone B3B4); marginal zone B cells: CD19^+^, GL7^-^, CD3e^-^, GR-1^-^, CD93^-^, CD138^-^, CD43^-^, F4/80^-^, CD21^hi^ and CD23^-^; B-1a cells: CD19^+^, CD43^+^ and CD5^+^ (clone 53-7.3); and B-1b cells: CD19^+^, CD43^+^ and CD5^-^.

### Generation of MDA-Modified APOB100

Malondialdehyde bis(dimethyl acetal) (122 mmol, Sigma Aldrich) was activated by incubation in 1 M HCl for 1 hour and quenched with the addition of 6 M NaOH until it equilibrated at pH 10. Activated MDA precipitate was washed with acetone and dried under vacuum. 0.5 mg APOB100 protein (Abcam) was incubated with 0.5 mg activated MDA for 24 hrs at 37°C and dialyzed into PBS.

### ELISA

Antibody isotype concentrations were detected using a 1:10,000 dilution of serum with a Mouse isotyping panel 1 kit (Meso Scale Diagnostics). Values for mouse IgG2a were not reported due to the C57BL/6J background containing the IgG2c allotype. Additionally, values for IgG3 and IgE were below detectable levels. Lipid-specific antibody levels were detected by coating polystyrene 96-well plates (Biolegend) with antigen containing 1 µg/mL of human LDL, MDA-modified human LDL (Academy Bio-Medical Co), APOB100 (Abcam), or MDA-modified APOB100 in coating buffer (100 µL/well; 35 mmol/L sodium carbonate, 68 mmol/L sodium bicarbonate) and incubated overnight at 4°C. Serum antibodies were analyzed at a dilution of 1:1000 for IgM and 1:200 for IgG1 diluted in 5% BSA and 1x PBS. Detection antibodies (goat anti-mouse IgM-HRP or goat anti-mouse IgG1-HRP, Southern Biotech) were added at 1:10,000 dilution, and signal was detected with TMB peroxidase substrate (Vector Labs). oxLDL serum levels were measured using a 1:20 dilution of serum with the mouse oxidized low density lipoprotein ELISA kit (Mybiosource) per manufacture’s recommendation. Plates were read using a SpectraMax M2 plate reader (Molecular Devices) at 450 nm.

### Multiplex Antigen Screen

Lipid antigens were screened using PIP Strips (Echelon Biosciences). Each membrane was incubated with a 1:100 dilution of serum and detected with a 1:10000 dilution of α-IgH+L-HRP (Southern Biotech). Signal was detected by addition of 5 mL TMB substrate (Vector Laboratories) per manufacturers recommendation. Protein antigens were determined through the utilization of a human proteome microarray (CDI Labs, HuProt Human Proteome Microarray, v 4.0) (29). Serum (1:1000 dilution) was added to the HuProt array and antibody binding identified after incubation with a fluorescent secondary antibody; Alexa647-anti-mouse-IgM and Cy3-anti-mouse-IgG. Fluorescence was detected and antibody-protein binding assessed, and specificity analyzed. Non-specific hits that directly bound to secondary antibodies were eliminated from the analysis of samples. CDI software was used to quantify the specificities of each sample to specific proteins on the array based on z-score. Z-score is the average of the duplicate spots of a given protein calculated as:

$$\text{IgM z}=\frac{\left(F635-{F635}_{avg}\right)}{{F635}_{std}}\;\text{and}\;\text{IgG z}=\frac{\left(F532-{F532}_{avg}\right)}{{F532}_{std}}$$

Significant targets were determined by averaging the z-score for each protein among all mice (*ApoE^-/-^*, n = 15; C57BL/6J, n = 10) with a cut-off of average z-score > 3. P-values were calculated by two-tailed T-test comparing *ApoE^-/-^* with C57BL/6J.

## Results

### *ApoE^-/-^ Aid^-/-^* and *ApoE^-/-^* Mice Had Similar Levels of Elevated Serum Cholesterol

To systematically examine the mechanisms of IgM and IgG on atherosclerosis, 12-wk old C57BL/6J, *ApoE^-/-^*, and *ApoE^-/-^ Aid^-/-^* mice were placed on a high-fat diet for 16 additional weeks (**Figure S1A**). We first determined the effects of AID deficiency on diet-induced hypercholesterolemia in the *ApoE^-/-^* background. Compared to C57BL/6J controls, *ApoE^-/-^* and *ApoE^-/-^ Aid^-/-^* mice had a significant 7.2-fold and 5.4-fold increase in total cholesterol levels, respectively (**Figure S1B**). Thus, the loss of AID function had little influence on total cholesterol accumulation in APOE-deficient mice, in that both strains had higher levels than C57BL/6J. These results reinforce the appropriateness of comparing the two *ApoE^-/-^* models for analyzing the effect of IgM *vs*. switched isotypes on atherosclerosis.

### Loss of Class-Switched Antibodies in *ApoE^-/-^ Aid^-/-^* Mice Blocked Atherosclerosis Development

**Figure 1:**
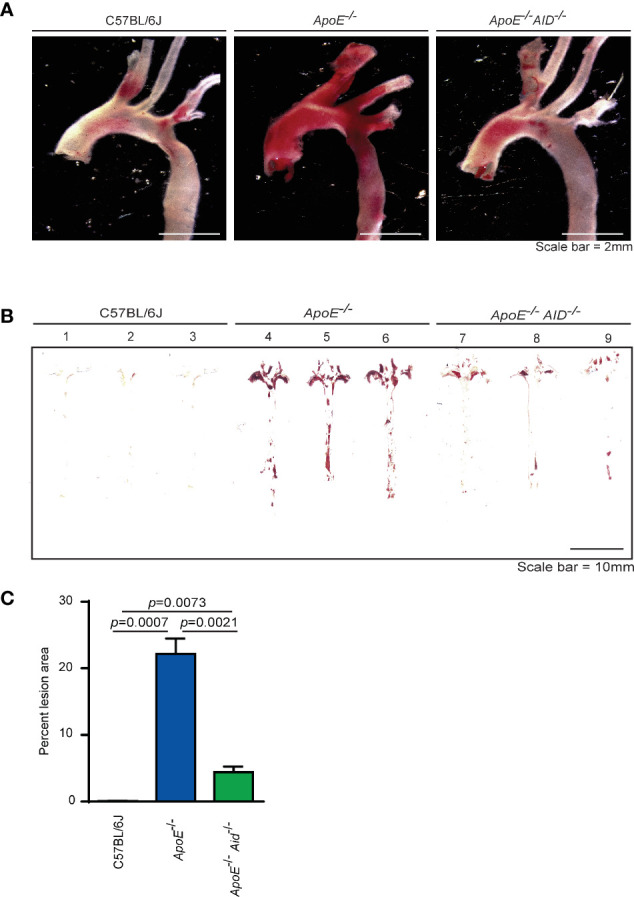
Lipid accumulation in aortas from mice fed a high fat diet for 4 months. **(A)** Representative images of Oil Red O-stained aortic arch and branches and **(B)** en face thoracic and abdominal aortas from 3 mice each from C57BL/6J, *ApoE^-/-^* and *ApoE^-/-^ Aid^-/-^* genotypes. **(C)** Quantification of Oil Red O staining area from en face images using HALO software. Error-bars represent standard deviation from a total of 11-15 mice per genotype. Statistics were calculated by two-tailed T-test.

In *ApoE^-/-^* mice, plaque development occurs at sites in which blood flow is disrupted. These areas include the aortic valves, aortic arch, and abdominal aorta. To examine the effects of class switching on plaque development, aortas from 28-wk old mice were stained with Oil Red O to analyze lipid accumulation. Consistent with previous reports (30–32), diet-induced atherosclerosis was prevalent in the aortic arch of *ApoE^-/-^* mice but not in C57BL/6J controls (**Figure 1A**). In striking contrast, the loss of class switching drastically reduced plaque levels in *ApoE^-/-^ Aid^-/-^* mice. This reduction occurred in both the aortic arch and the abdominal aorta (**Figures 1A, B**). In addition to total reduction in staining, *ApoE^-/-^ Aid^-/-^* mice also had reduced plaque volume, with smaller expansion into the vessel lumen (**Figure S2**). Quantification of plaque staining showed that *ApoE^-/-^* mice displayed detectable levels of Oil Red O staining in 22% of the aortic surface, while *ApoE^-/-^ Aid^-/-^* mice had significantly reduced staining covering only 4% of the total aorta (**Figure 1C**). These data robustly demonstrate that inhibiting antibody diversification substantially altered atherosclerosis even in the presence of hypercholesterolemia.

### Atherosclerosis Altered B Cell Subsets in the Aorta but Not Spleen or Peritoneum

**Figure 2:**
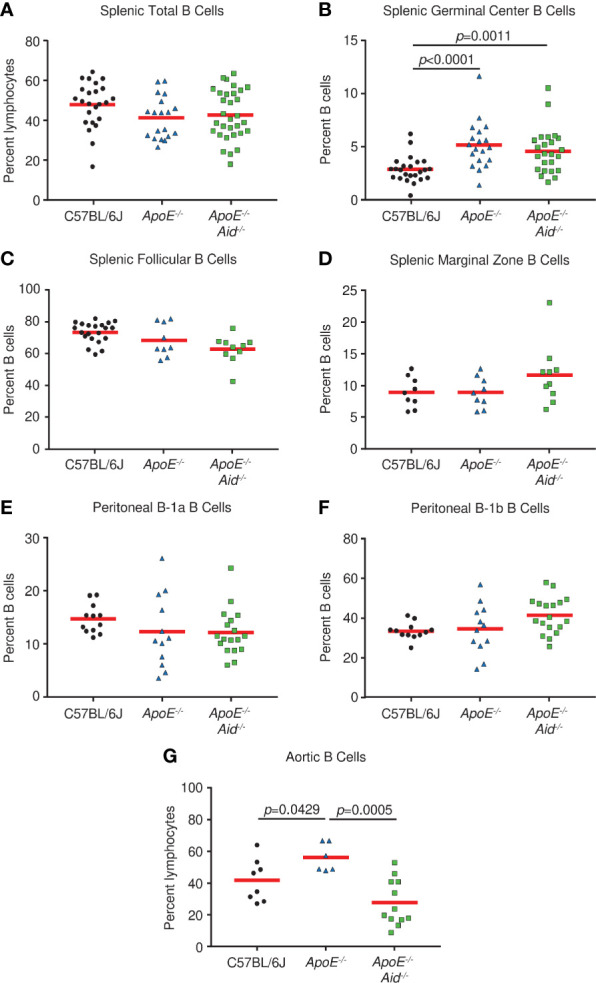
B cell subsets in spleen, peritoneal cavity, and aorta from C57BL/6J, *ApoE^-/-^* and *ApoE^-/-^ Aid^-/-^* mice. **(A)** Total, **(B)** germinal center, **(C)** follicular, and **(D)** marginal zone B cells were analyzed from spleens of mice at 28 weeks. **(E)** B1a and **(F)** B-1b cells were isolated from the peritoneal cavity. **(G)** Aortic B cells were isolated from the aorta and adjacent fat layer. Symbols represent percentages in individual mice with red line displaying the mean. *p*-values represent two-tailed T-test.

It has been speculated that the severity of atherosclerosis can be modulated by altering specific populations of B cells. For example, it was reported that splenic marginal zone B cells increased plaque development when depleted (33), peritoneal B-1a cells either promoted atherosclerosis when cells were depleted or inhibited plaque size when cells were expanded (6, 7, 34), and a novel splenic subset termed IRA B cells which express GM-CSF was associated with the disease (35). Therefore, we systematically examined alterations in lymphocyte populations from C57BL/6J, *ApoE^-/-^*, and *ApoE^-/-^ Aid^-/-^* mice on the high-fat diet. The splenic populations had the same percentage of total B cells (**Figure 2A**). Germinal center B cells were significantly increased in *ApoE^-/-^* and *ApoE^-/-^ Aid^-/-^* mice, suggesting that the elevated serum cholesterol in both strains (**Figure S1**) could activate B cells systemically (**Figure 2B**). Examination of splenic B cells revealed no statistical changes in follicular and marginal zone cells between the three strains (**Figures 2C, D**). We were unable to evaluate IRA B cells, since they only appeared in appreciable amounts in mice with severe dermatitis, which were excluded from the study. Notably, the B-1a and B-1b populations in the peritoneal cavity had similar levels among the three strains, and were not significantly increased in *ApoE^-/-^ Aid^-/-^* mice (**Figures 2E, F**).

Although rampant atherosclerosis in the arteries from *ApoE^-/-^* mice did not appear to alter B cell populations from spleen or peritoneum, it may affect B cells at the site of inflammation. B cells have recently been identified in the adipose tissue surrounding the aorta (3); therefore we examined the aorta and surrounding fatty tissue for lymphocyte content. Compared to C57BL/6J controls, *ApoE^-/-^* mice had increased numbers of B cells (**Figure 2G**). However, this phenotype was absent in *ApoE^-/-^ Aid^-/-^* mice, which shows that atherosclerosis itself was associated with local B cell expansion. Our data demonstrates that atherosclerosis in *ApoE^-/-^* mice affected B cell content on the aorta and not in the spleen, whereas serum hypercholesterolemia in *ApoE^-/-^* and *ApoE^-/-^ Aid^-/-^* mice appeared to systemically activate splenic germinal center B cells.

### High IgM Serum Titers to MDA-oxLDL Were Found in *ApoE^-/-^ Aid^-/-^* Mice but Not *ApoE^-/-^* Mice

**Figure 3:**
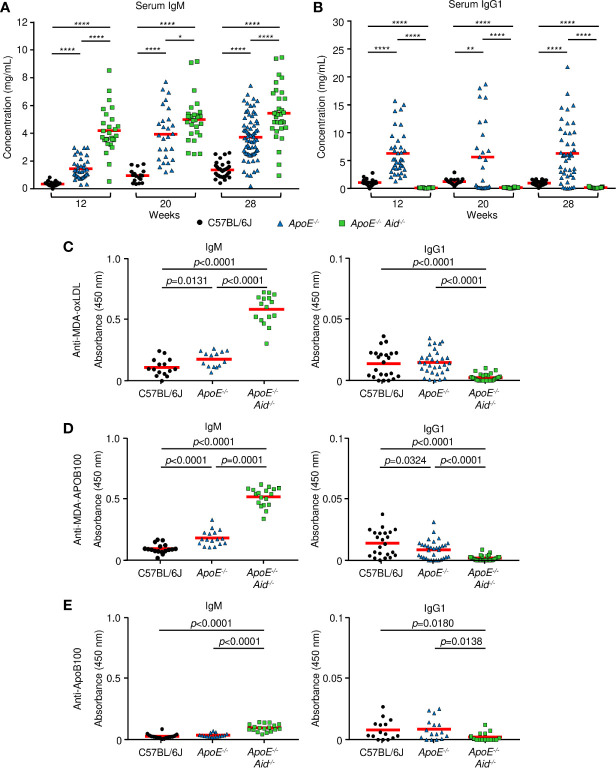
Serum antibody concentrations from mice. **(A, B)** total immunoglobulin measured by ELISA at 12, 20 and 28 weeks on a high fat diet. **(A)** IgM and **(B)** IgG1 from C57BL/6J (black circle), *ApoE^-/-^* (blue triangle) or *ApoE^-/-^ Aid^-/-^* (green square) mice. Red line represents the mean from 17 or more independent mice. *0.05 > p > 0.01, **0.01 > p > 0.001, ***0.001 > p > 0.0001, ****p < 0.0001. **(C–E)**, IgM and IgG1 antibody titers measured by ELISA at 28 weeks on the diet. **(C)** MDA-oxLDL, **(D)** MDA-ApoB100, and **(E)** ApoB100. IgM sera was diluted 1:1000, and IgG1 sera was diluted 1:200 for analysis. Symbols represent percentages in individual mice with red line displaying the mean from 14-16 mice per genotype. Significance was determined by two-tailed T-test.

To test the hypothesis that IgM and IgG antibodies function in opposing roles during atherosclerosis, we examined the effects of hypercholesterolemia and AID deficiency on IgM and IgG titers. Sera was collected at the start of the high-fat diet at 12 weeks of age, midway at 20 weeks, and the end at 28 weeks. Immunoglobulin concentrations were measured by multiplex ELISA for IgM, IgG1, IgG2b, and IgA (**Figures 3A, B**, **S3A, B**). For IgM levels (**Figures 3A**, **S3C**), C57BL/6J mice increased from 0.34 mg/mL to 1.35 mg/mL, and *ApoE^-/-^* mice expanded from 1.46 to 3.72 mg/mL. This indicates that diet and the resulting development of atherosclerosis boosts IgM titers, presumably by reacting to cholesterol epitopes. In the absence of AID, IgM titers were substantially elevated by the inability to class switch, as previously seen in *Aid^-/-^* mice (36) and humans with hyper-IgM syndrome (37). Consistent with this genetic block, *ApoE^-/-^ Aid^-/-^* had 4.20 mg/mL IgM prior to the start of the high-fat diet, which increased to 5.45 mg/mL.

For the switched isotypes, IgG1 is the predominant isotype expressed in *ApoE^-/-^* serum, relative to IgG2b and IgA. For IgG1 levels, C57BL/6J mice had consistent levels of about 1 mg/mL throughout the diet, and *ApoE^-/-^* mice had consistent levels of around 6 mg/mL (**Figure 3B**). Thus, at multiple time points, *ApoE^-/-^* mice had very high levels of IgG1 compared to C57BL/6J mice. As expected, *ApoE^-/-^ Aid^-/-^* mice had no IgG1. IgG2b levels were relatively constant at about 1.5 mg/mL in C57BL/6J and *ApoE^-/-^* sera from 12-28 weeks (**Figures S3A, C**). IgA levels were constant in C57BL/6J mice at 0.14 mg/mL and were slightly elevated in *ApoE^-/-^* mice at around 0.5 mg/mL (**Figures S3B, C**). *ApoE^-/-^ Aid^-/-^* mice had only background levels of class-switched serum antibodies (**Figure S3C**). This serum analysis shows that in *ApoE^-/-^* mice, both IgM and IgG1 were elevated, yet the kinetics show that high IgG1 titers occurred prior to the high-fat diet while IgM titers were further boosted by the diet. The observation that both the *ApoE^-/-^ Aid^-/-^* and *ApoE^-/-^* mice have elevated IgM titers, suggests a more complex dynamic where IgM titers by themselves are insufficient at controlling atherosclerosis.

Do IgM antibodies from *ApoE^-/-^ Aid^-/-^* mice bind different antigens than IgM antibodies from *ApoE^-/-^* mice? To examine antigen specificity, we determined if serum antibodies could bind to MDA-oxLDL, which has been implicated in protection during atherosclerosis (38). IgM titers to MDA-oxLDL were modestly elevated in *ApoE^-/-^* serum compared to C57BL/6J controls (**Figure 3C**). Strikingly, the MDA-oxLDL specific antibody titers were further increased by 3-fold in the *ApoE^-/-^ Aid^-/-^* mice (**Figure 3C**). In contrast, only modest and similar levels of IgG1 to MDA-oxLDL was seen in C57BL/6J and *ApoE^-/-^* serum (**Figure 3C**), consistent with previous observations (26, 39). This indicates that although the *ApoE^-/-^* mice had high levels of circulating IgG1, it was directed against different antigens than those recognized by IgM from *ApoE^-/-^ Aid^-/-^* mice. Similar results were observed for IgM and IgG1 binding to MDA-modified APOB100 protein, suggesting the antibody response is directed against the protein component of LDL (**Figure 3D**). These antibodies were specific for the oxidized epitope, as they only bound at low levels to unmodified ApoB100 (**Figure 3E**) or LDL (data not shown). To test whether the different levels of MDA-LDL antibodies were due to altered oxLDL levels in each strain of mice, we analyzed serum oxLDL concentrations. *ApoE^-/-^* mice had the highest levels of oxLDL at 88.4 ng/mL while *ApoE^-/-^ Aid^-/-^* mice had 84.0 ng/mL and C57BL/6J had only 75 ng/mL. Thus, the differences in antibody specificity were not a result of lower antigen exposure in *ApoE^-/-^* mice.

### *ApoE^-/-^* Mice Made IgM and IgG Antibodies to Self-Proteins

**Figure 4:**
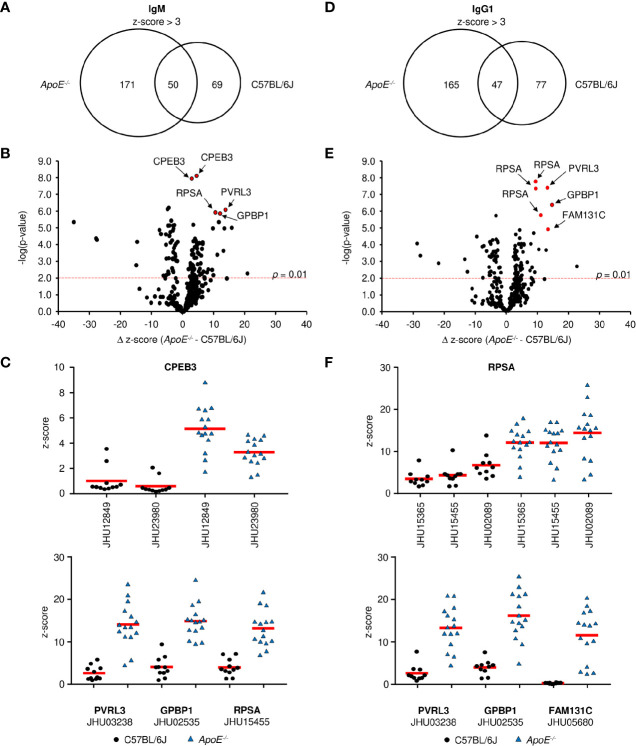
Antigen identification in serum from *ApoE^-/-^* and C57BL/6J mice. **(A, D)** Venn diagrams showing the overlap of **(A)** IgM and **(D)** IgG1 antigen targets in *ApoE^-/-^* and C57BL/6J mice. Volcano plots examined the change in z-score between targets in *ApoE^-/-^* and C57BL/6J mice and the relative significance of those differences for **(B)** IgM and **(E)** IgG1. Red dashed line represents a *p*-value of 0.01 (two-tailed T-test). **(C, F)** Z-score values for representative antigens identified by red circles in **(B, E)** in all individual mice for **(C)** IgM and **(F)** IgG1. Values were calculated from 10 C57BL/6J and 15 *ApoE^-/-^* mice. Red solid line represents mean value.

When the modest levels of MDA-specific antibodies are compared to the drastic elevation of total serum Ig in *ApoE^-/-^* mice, it becomes clear that other antigens are driving most B cell responses during plaque formation. To identify these antigens, we first looked at antibodies specific to phospholipids as they have been suggested to play a role in atherosclerosis (40). Analysis of 15 different lipid molecules produced no significant signal indicating that the majority of antibodies are not against lipids (data not shown). Due to the established link between autoimmune disorders and heart disease (41), we then investigated whether these antibodies were generated against self-proteins on a microarray containing 81% of the human proteome. To control for cross-species binding, serum was tested from the C57BL/6J high-fat diet control mice. Analysis of 23,120 protein targets showed that IgM from *ApoE^-/-^* mice significantly bound (average z-score > 3) 221 proteins (**Data File S1**), while IgM from C57BL/6J mice bound 120 proteins (**Data File S2**), with an overlap of 50 (**Figure 4A**). To identify proteins which were specific to *ApoE^-/-^* IgM, the δ z-score values was calculated for all targets which displayed significant IgM binding in either *ApoE^-/-^* or C57BL/6J serum (291 proteins). Plotting δ z-score vs -log(*p*-value) showed that there were 121 targets that were significantly different (*p* < 0.01), of which 75 bound to more IgM in the *ApoE^-/-^* serum compared to C57BL/6J (**Figure 4B**). One target, Cytoplasmic polyadenylation element-binding protein 3 (CPEB3), appeared multiple times, suggesting antibody binding to conserved regions of the various CPEB3 isoforms on the array, further verifying accuracy of the technique. Furthermore, analysis of z-score values from individual mice confirmed that levels of IgM antibodies to CREB3, Nectin-3 (PVRL3), Vasculin (GPBP1), and 40S ribosomal protein SA (RPSA), were consistently higher in *ApoE^-/-^* serum compared to C57BL/6J serum (**Figure 4C**). Similar analysis of IgG1 protein targets revealed 212 significant proteins for *ApoE^-/-^* IgG1 (**Data File S3**) and 124 for C57BL/6J IgG1 (**Data File S4**), with 47 shared proteins (**Figure 4D**). Analysis of δ z-score vs -log(*p*-value) values for the 289 proteins identified, 99 were significantly different between the two strains. 59 targets were found more often in *ApoE^-/-^* serum, including RSPA, PVRL3, GPBP1, and Family with sequence similarity 131 member C (FAM131c), and were consistently higher when looking at individual mice (**Figures 4E, F**). To examine if *ApoE^-/-^ Aid^-/-^* mice also produced self-antibodies, we analyzed IgM z-scores for the top *ApoE^-/-^* targets identified in **Figure 4B**. Compared to C57BL/6J serum, *ApoE^-/-^ Aid^-/-^* IgM antibodies do display elevated binding to these antigens, but they are lower than the *ApoE^-/-^* serum, consistent with reduced plaque burden in the absence of AID (**Figure S4**).

**Figure 5:**
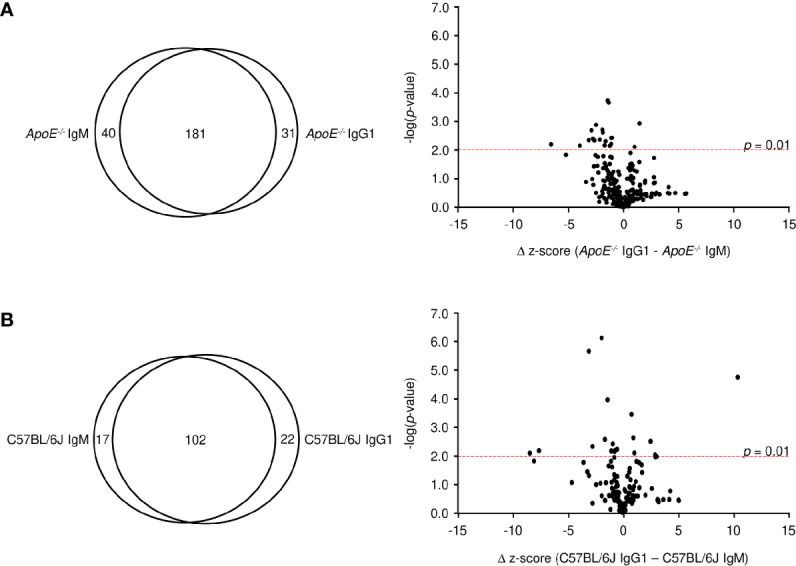
Comparative analysis of IgM and IgG1 antibodies. Venn diagram and volcano plots comparing significant protein targets for IgM and IgG within **(A)** *ApoE^-/-^* and **(B)** C57BL/6J serum. Red dashed line represents a p-value of 0.01 determined by two-tailed T test.

The observation that the *ApoE^-/-^* IgM and IgG1 protein targets identified similar proteins (i.e. RPSA, PVRL3, GPBP1) suggests that the B-2 antibody response is not isotype specific. To address this, we analyzed average z-scores for all protein targets and found that 72% of targets (z-score > 3) are shared between IgM and IgG1 for *ApoE^-/-^* mice (**Figure 5A**), with only 20 targets showing statistically significant differences (*p* < 0.01). The observation is also found with C57BL/6J mice where 72% of all targets are shared between IgM and IgG1 (**Figure 5B**) and only 18 being statistically different. These results confirm that in atherosclerotic mice, antibody responses are generated against self-proteins and are independent of isotype.

## Discussion

To characterize the role of B cells and antibodies in atherosclerosis, we generated *ApoE^-/-^ Aid^-/-^* mice which develop hypercholesterolemia and are deficient in generating class-switched antibodies. In the absence of switched isotypes, these mice had dramatically less arterial plaque than their *ApoE^-/-^* counterparts. Previous experiments have shown that germline-encoded IgM produced by B-1 cells is protective (6, 7). By increasing the presence of B-1a or B-1b cells in a mouse, through adoptive transfer or genetic manipulation, oxLDL-specific IgM antibody titers were increased and disease was reduced. However, in our study, B-1 cells were not increased in *ApoE^-/-^* mice compared to C57BL/6J mice. More importantly, the protective MDA-oxLDL specific IgM titers only modestly increased in *ApoE^-/-^* compared to C57BL6J mice and were insufficient at controlling disease development with the increased lipid content. Thus, our data imply that B-1 cells are providing a basal level of antibody protection, but are unable to produce sufficient levels of antibody to control hyperlipidemia. In contrast, IgM serum levels were greatly raised in *ApoE^-/-^ Aid^-/-^* mice, with elevated titers of antibody against MDA-oxLDL and its protein component MDA-APOB100, suggesting the B-2 cell response can provide protection when directed toward the correct antigen. However, in *ApoE^-/-^* mice, IgM and IgG1 did not bind to MDA-oxLDL at high levels but bound to numerous self-proteins, presumably increasing inflammation and promoting atherosclerosis consistent with previous studies (12, 13). These antibodies were detected against intracellular proteins, suggesting the environment of the plaque is driving the B cell adaptive response, consistent with our observation of increased B cells around the aorta.

These data support a hierarchy in appearance of antibody specificity to their cognate antigens, where the balance between oxLDL and oxLDL-specific antibodies is a key regulator of disease progression. When MDA-oxLDL specific antibody titers are low (as in *ApoE^-/-^* mice) they cannot block oxLDL uptake by macrophages and plaques develop (9, 18). Once established, macrophages convert to foam cells and rupture, releasing self-proteins which could be oxidized/modified in the necrotic environment, and may be the source of the elusive epitopes which elevate IgM and IgG1 titers in *ApoE^-/-^* mice. This theory is consistent with data showing a correlation between autoimmune diseases which generate high levels of self-antibodies and which develop accelerated atherosclerosis (41). B cells specific for these antigens expand and produce autoantibodies, which may bind to Fc*γ* receptors on macrophages (16–19) and vascular smooth muscle cells (16, 20), to release inflammatory cytokines and set up a virtual war zone in the artery. It will be interesting to see if the autoantibodies against a specific protein (i.e., CPEB3 or RPSA) promote a particular inflammatory response in the aorta, or if any self-protein or combination of self-proteins can generate inflammation and plaque formation.

Why do *ApoE^-/-^ Aid^-/-^* mice produce high titers of MDA-oxLDL specific antibodies? In addition to CSR deficiency, the mice also have no somatic hypermutation, causing only germline-encoded antibodies to be expressed, which may bind to the lipids with sufficient affinity (6). Although *ApoE^-/-^* mice produce a low level of MDA-oxLDL antibodies, it is possible that mutated antibodies drift away from binding MDA-oxLDL and decrease the response. Alternatively, during atherosclerosis, more prominent self-antigens (i.e., RPSA) are released to hijack the immune response and drive the generation of high-affinity auto-antibodies. Both possibilities may not occur in *ApoE^-/-^ Aid^-/-^* mice due to the absence of SHM.

In conclusion, we show that high levels of oxLDL antibodies prevent atherosclerosis, consistent with recent experiments showing an antigen-specific Fab molecule can regulate plaque formation (40). However, in *ApoE^-/-^* mice, the oxLDL antibody response is inadequate and the response is directed towards self-antigens potentially coming from the plaque environment. Strategies which boost antibody responses to MDA-oxLDL or block self-antigen recognition, could prevent lipid accumulation and decrease atherosclerosis.

## Data Availability Statement

The original contributions presented in the study are included in the article/**Supplementary Material**. Further inquiries can be directed to the corresponding author.

## Ethics Statement

All animal protocols were reviewed and approved by the Animal Care and Use Committee of the National Institute on Aging.

## Author Contributions

The concept was conceived by MW, EL, PG, and RM. The overall study design was developed by MH, H-SP, and RM. Additional experiments were performed by KZ, JA-G, JZ, LZ, and RT. Manuscript was written by MH, PG, and RM. All authors contributed to the article and approved the submitted version.

## Funding

This work was supported entirely through the Intramural Research Program at the National Institutes of Health (NIH), National Institute on Aging (AG000777). The authors have no disclosures to report.

## Conflict of Interest

The authors declare that the research was conducted in the absence of any commercial or financial relationships that could be construed as a potential conflict of interest.
