# Supplementary figures and images for: Auto-Antibody Production During Experimental Atherosclerosis in ApoE-/- Mice
Source: Front Immunol. 2021 Jul 9;12:695220. doi: 10.3389/fimmu.2021.695220 (PMC8299997; doi:10.3389/fimmu.2021.695220)

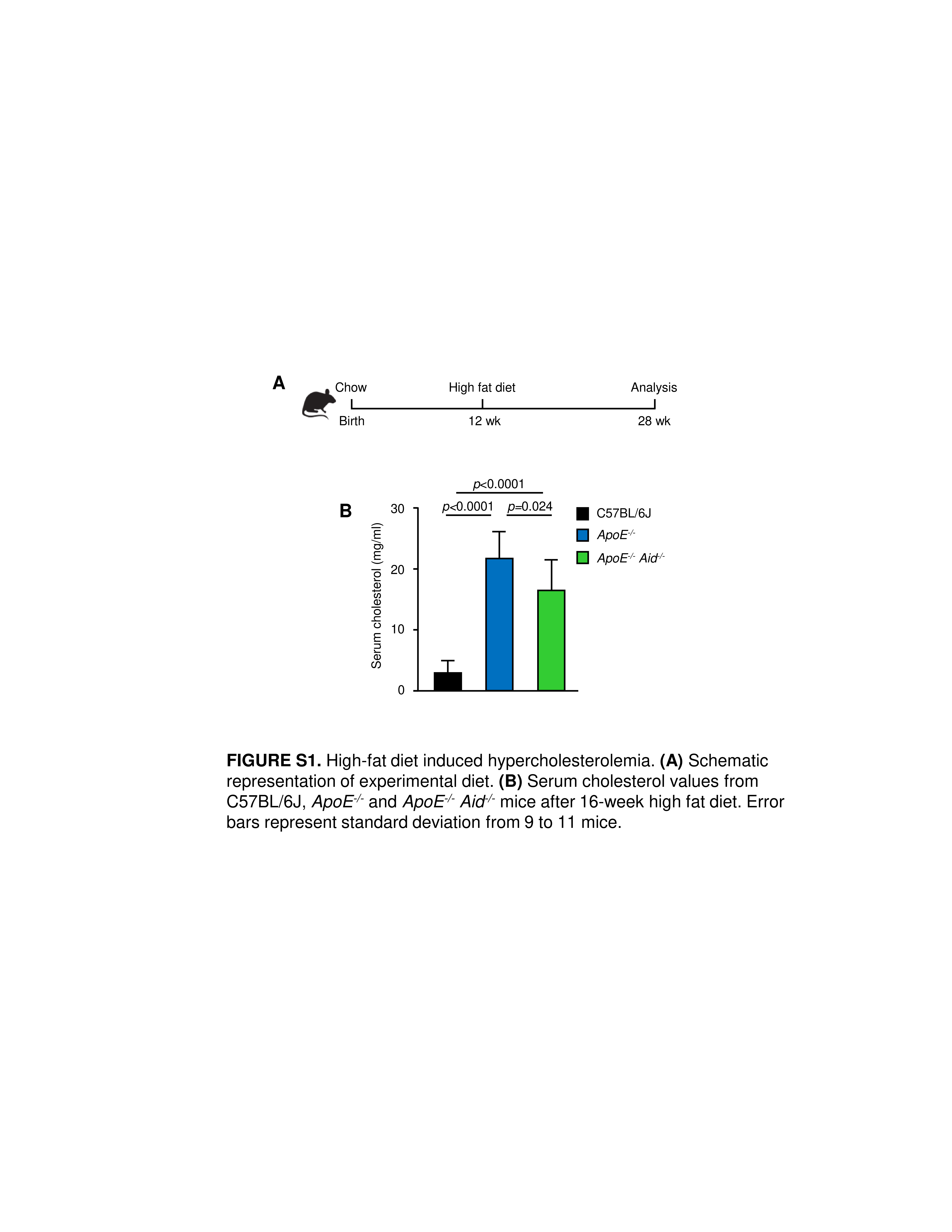

Supplement: Supplementary file 2 [file Image_1.tif]

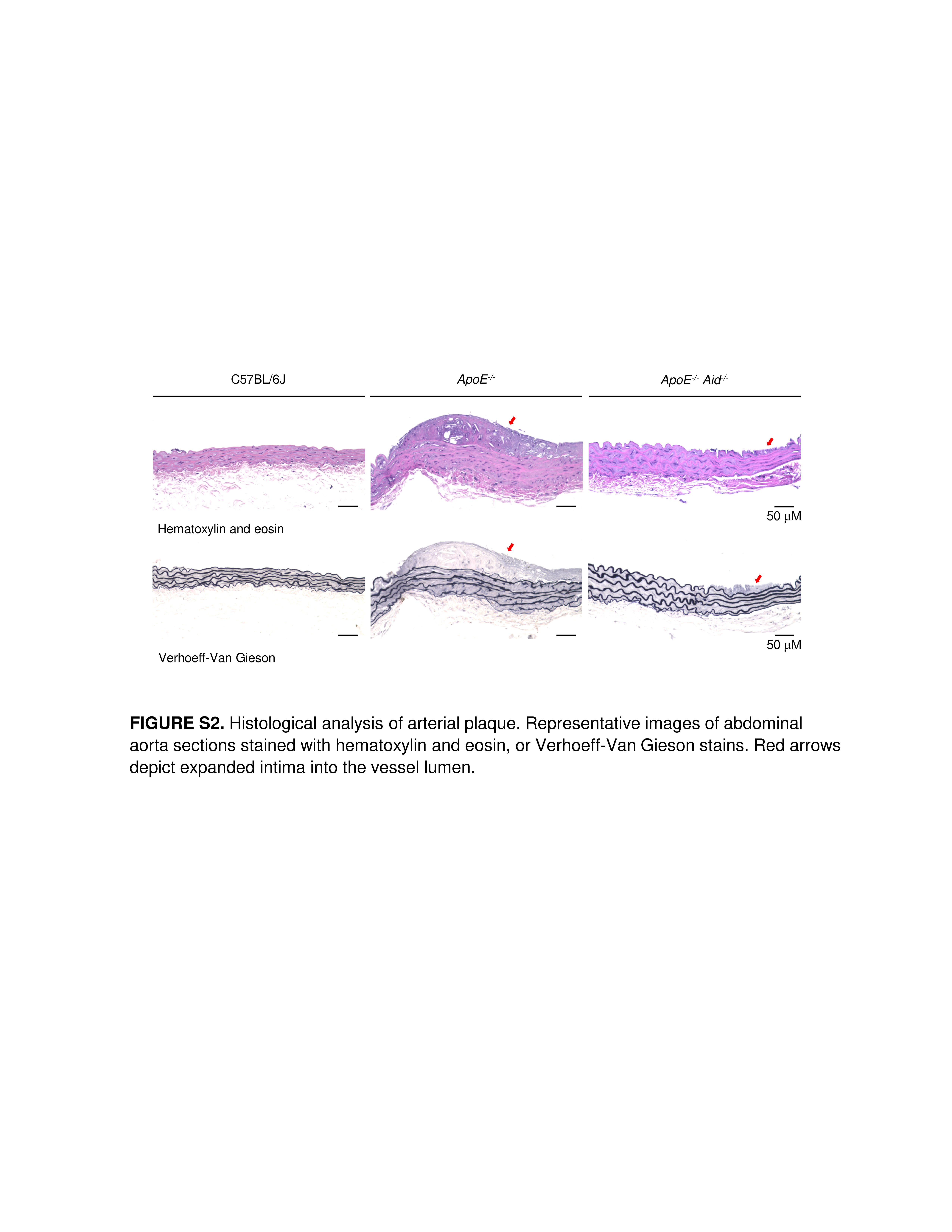

Supplement: Supplementary file 3 [file Image_2.tif]

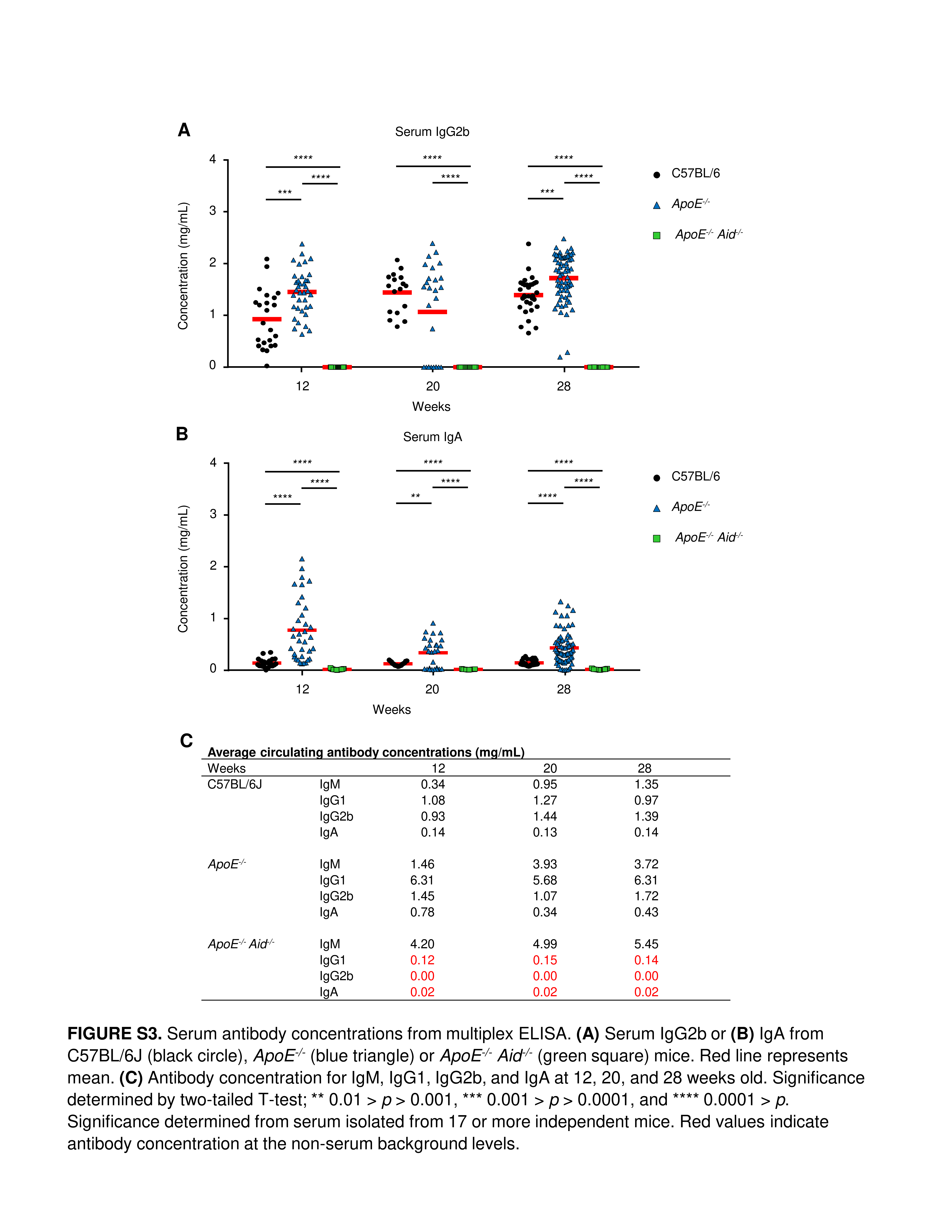

Supplement: Supplementary file 4 [file Image_3.tif]

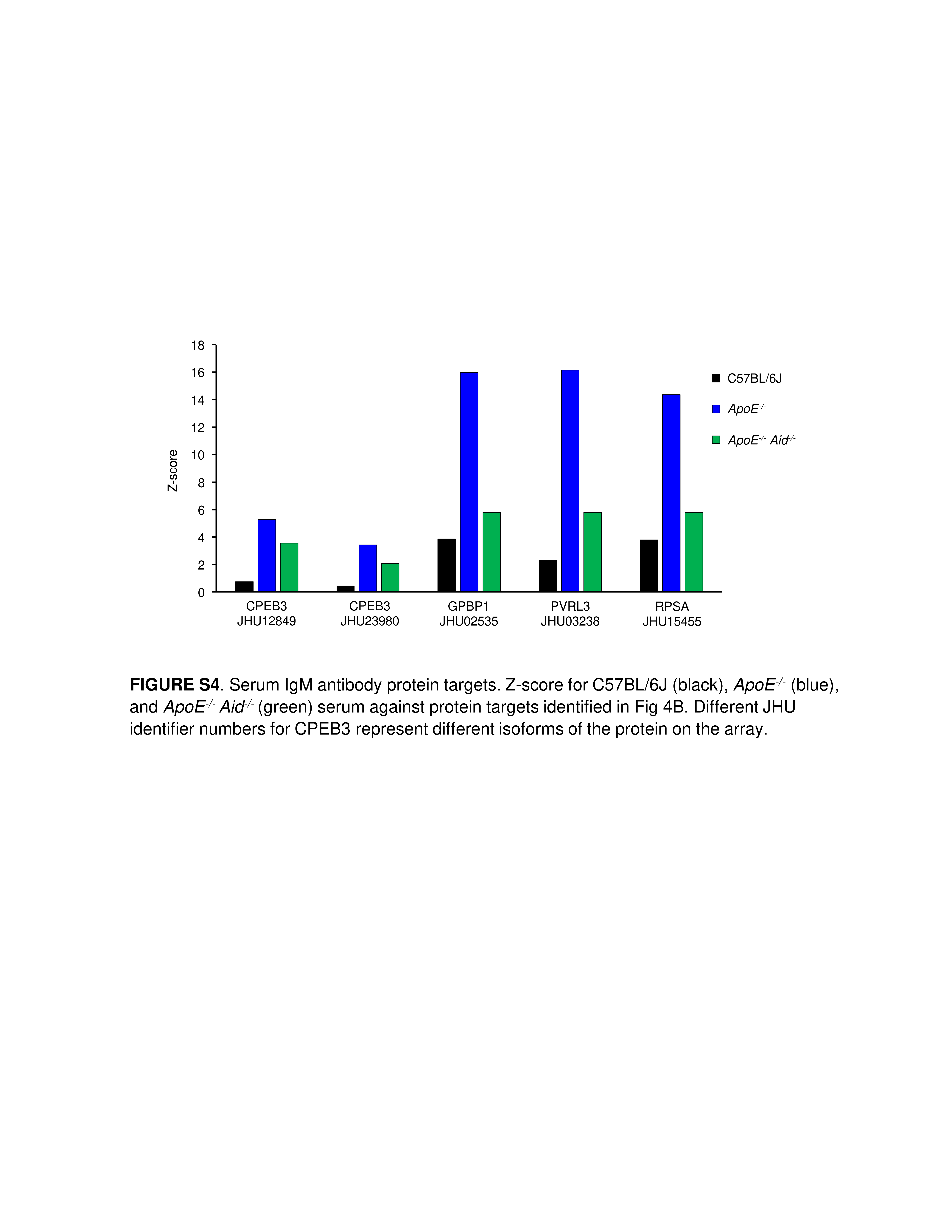

Supplement: Supplementary file 5 [file Image_4.tif]
